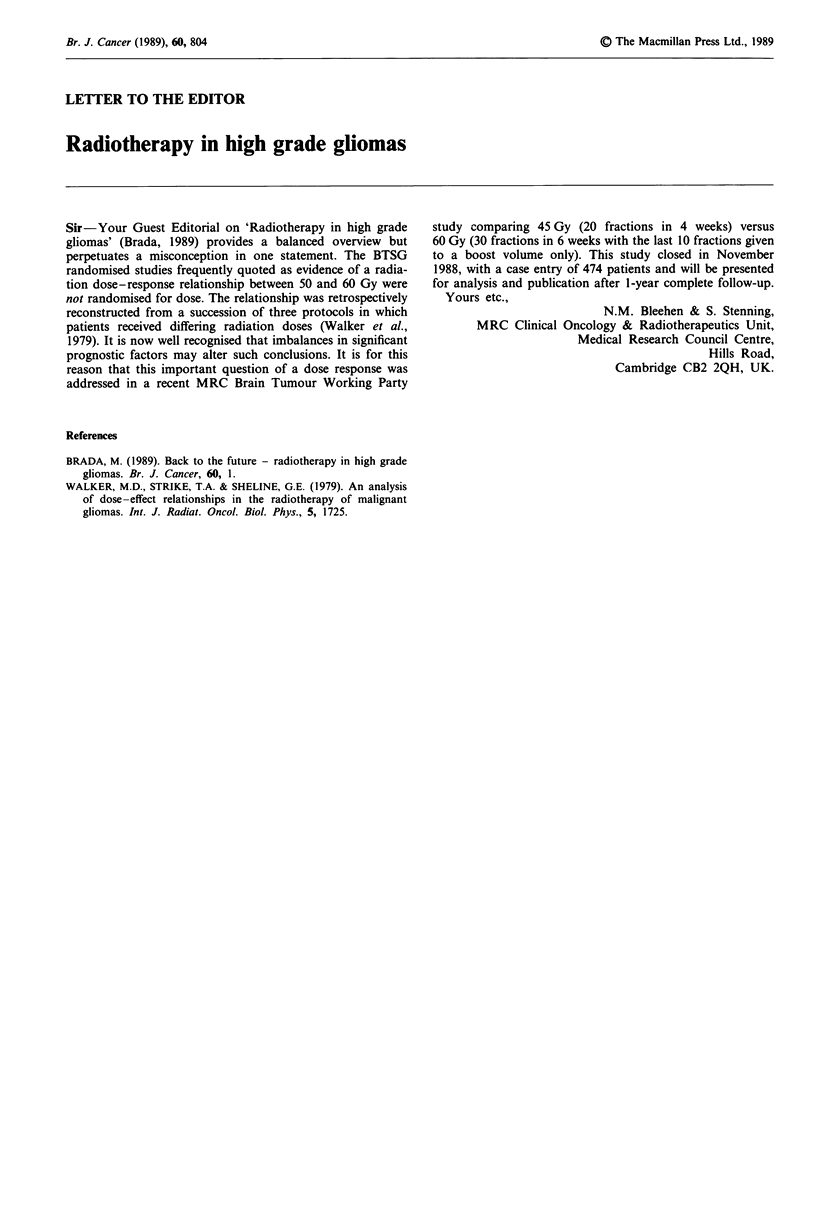# Radiotherapy in high grade gliomas

**Published:** 1989-11

**Authors:** N.M. Bleehen, S. Stenning


 © The Macmillan Press Ltd., 1989

### LETTER TO THE EDITOR

### Radiotherapy in high grade gliomas

**Sir**—Your Guest Editorial on 'Radiotherapy in high grade gliomas' (Brada, 1989) provides a balanced overview but perpetuates a misconception in one statement. The BTSG randomised studies frequently quoted as evidence of a radiation dose-response relationship between 50 and 60 Gy were *not* randomised for dose. The relationship was retrospectively reconstructed from a succession of three protocols in which patients received differing radiation doses (Walker *et al.*, 1979). It is now well recognised that imbalances in significant prognostic factors may alter such conclusions. It is for this reason that this important question of a dose response was addressed in a recent MRC Brain Tumour Working Party study comparing 45 Gy (20 fractions in 4 weeks) versus 60 Gy (30 fractions in 6 weeks with the last 10 fractions given to a boost volume only). This study closed in November 1988, with a case entry of 474 patients and will be presented for analysis and publication after 1-year complete follow-up.

Yours etc.,

N.M. Bleehen & S. Stenning,
MRC Clinical Oncology & Radiotherapeutics Unit,
Medical Research Council Centre,
Hills Road,
Cambridge CB2 2QH, UK.

### References

BRADA, M. (1989). Back to the future – radiotherapy in high grade gliomas. *Br. J. Cancer*, **60**, 1.

WALKER, M.D., STRIKE, T.A. & SHELINE, G.E. (1979). An analysis of dose-effect relationships in the radiotherapy of malignant gliomas. *Int. J. Radiat. Oncol. Biol. Phys.*, **5**, 1725.